# Sialic acid: an attractive biomarker with promising biomedical applications

**DOI:** 10.2478/abm-2022-0020

**Published:** 2022-08-31

**Authors:** Aida Doostkam, Leila Malekmakan, Alireza Hosseinpour, Sahar Janfeshan, Jamshid Roozbeh, Fatemeh Masjedi

**Affiliations:** Shiraz Nephro-Urology Research Center, Shiraz University of Medical Sciences, Shiraz 7193635899, Iran; Student Research Committee, Shiraz University of Medical Sciences, Shiraz 7134853185, Iran

**Keywords:** cardiovascular diseases, diabetic nephropathies, molecular targeted therapy, neoplasms, neuraminic acids, sialic acid, virus diseases

## Abstract

This broad, narrative review highlights the roles of sialic acids as acidic sugars found on cellular membranes. The role of sialic acids in cellular communication and development has been well established. Recently, attention has turned to the fundamental role of sialic acids in many diseases, including viral infections, cardiovascular diseases, neurological disorders, diabetic nephropathy, and malignancies. Sialic acid may be a target for developing new drugs to treat various cancers and inflammatory processes. We recommend the routine measurement of serum sialic acid as a sensitive inflammatory marker in various diseases.

Sialic acid was discovered in the 20th century in bovine submaxillary mucin, and its family contains more than 50 members [1]. The current narrative review considers sialic acid as a family of 9-carbon-containing monosaccharides.

Sialic acids form the terminus of glycoproteins, glycolipids (specifically in the nervous system), and oligosaccharides, which are widely distributed on vertebrate cell surfaces. They are neuraminic acid derivatives that modulate various physiological and pathological processes. The most familiar member of this group is *N*-acetylneuraminic acid (Neu5Ac or NANA) [2] (**Figure 1A**).

**Figure 1:**
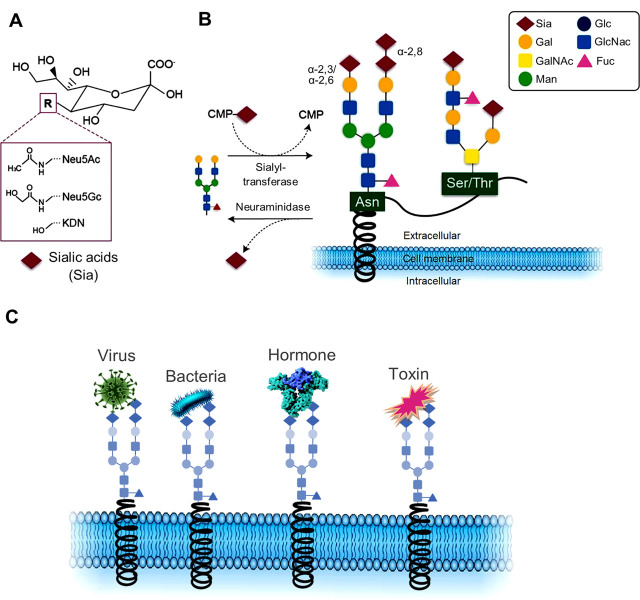
**(A)** Chemical structure of the 3 main sialic acids, *N*-acetylneuraminic acid (Neu5Ac), *N*-glycolylneuraminic acid (Neu5Gc), and deaminoneuraminic acid (KDN). **(B)** Sialic acids are transferred onto acceptor glycans via sialyl transferase enzymes that use the activated cytidine-5′-monophosphate-sialic acid (CMP-Sia) as a donor molecule. Sialyl residues may be added terminally to galactose residues in α-2,3 or α-2,6 linkage, or to sialic acid residues in α-2,8 connection. Such glycan chains may be attached to glycoproteins via asparagine (*N*-glycan) or serine or threonine residues (*O*-glycans). Sialic acids may be released via hydrolytic enzymes called neuraminidases. **(C)** Examples of pathobiological interactions involving sialic acids with viruses, bacteria, hormones, and toxins. Gal, galactose; GalNAc, *N*-acetylgalactosamine; Man, mannose; Glc, glucose; GlcNAc, *N*-acetylglucosamine; Fuc, fucose.

Although sialic acid is widely expressed on the plasma membrane of normal cells, it is upregulated in some pathological conditions, including cancers, infections, neurological disorders, cardiovascular diseases (CVD), and diabetic nephropathy [3, 4]. Serum sialic acid level is correlated with the severity of various diseases, and it may provide a marker for inflammation, disease severity, and tumor progression. However, this needs to be further elucidated, and more studies must be conducted to demonstrate the possibility of sialic acid as a general inflammatory marker [5].

Some studies have investigated sialic acid effects in various pathological conditions, and so we explored the role of sialic acid in the pathophysiology of neoplasms and other diseases.

## Search methodology

For this narrative review, 2 coauthors (AD and AH) searched electronic databases, including PubMed, Scopus, Web of Science, and Google Scholar, to find the relevant articles with time limitations (1991–2022). The keywords searched for were “sialic acid”, “neuraminic acids”, “viral infections”, “cardiovascular diseases”, “diabetic nephropathy”, “cancer”, “malignancy”, “serum marker”, “drug development”, and “drug target”. Then, these articles were screened based on their titles. All reviews, original articles, and clinical trial articles only in English were included. After finding the articles, each was screened to exclude irrelevant and duplicate studies. Next, the relevant articles were reviewed carefully, and the references were searched to find more articles.

Among a total of 2478 articles that were identified in databases, 131 full-text articles were chosen for careful review. Of the 131 studies we reviewed, 22 were regarding sialic acids as viral receptors [5,6,7,8,9,10,11,12,13,14,15,16,17,18,19,20,21,22,23,24,25,26], and 29 were regarding immune system evasion of cancers through sialic acid modification [27,28,29,30,31,32,33,34,35,36,37,38,39,40,41,42,43,44,45,46,47,48,49,50,51,52,53,54,55]. Sialic acids and CVD were indicated in 12 articles [56,57,58,59,60,61,62,63,64,65,66,67], while 8 highlighted sialic acid as a predictor of diabetic complications [4, 68,69,70,71,72,73,74]. There were 9 articles regarding the role of sialic acids in the central nervous system [75,76,77,78,79,80,81,82,83]. Sialic acid as a promising target for novel therapeutic drugs was pursued in 12 studies [26, 41, 82, 84,85,86,87,88,89,90,91,92].

## Sialic acids

*N*-Acetylneuraminic acid (Neu5Ac) is the most abundant member of the sialic acid family in mammals and is considered an α-keto acid (keto-aldononulosonic acid) [1, 2]. It is made in the eukaryotic cell cytoplasm and is activated by binding to cytosine 5′-monophosphate (CMP) in the nucleus and forming CMP-Neu5Ac. Next, CMP-Neu5Ac is transferred to the end of glycoconjugates by sialyltransferases in the Golgi apparatus and consequently expressed on the cell membrane [2] (**Figure 1B**).

Postglycosylation modifications of sialoglycoconjugates help Neu5Ac increase sialic acid diversity. These modifications include *O*-acetylation, *O*-methylation, sulfation, phosphorylation, and lactylation [1]. Approximately 70% of cellular sialic acid concentration is expressed on the plasma membrane, and the rest is predominantly found in the smooth endoplasmic reticulum [3]. Moreover, sialic acids can be detected in body fluids such as serum, urine, breast milk, and human tissues, including the stomach, intestines, and salivary glands. Sialic acids are bound to plasma proteins such as fibrinogen, haptoglobin, and transferrin. They are also widely distributed in the erythrocytes, leukocytes, platelets, and vascular endothelial cells [2]. The fundamental role of the sialic acid family in many physiological and pathological processes, including cellular recognition and communication, cellular development, mediating bacterial and viral infection, tumor growth and metastasis, and immunology, has been well established [3]. Sialic acids can have contradictory roles. First, they can function as antirecognition agents by masking normal cellular recognition sites and dampening the immune system. Furthermore, they can act as a ligand for many molecules such as lectins, antibodies, viruses, and bacteria by facilitating cell recognition and adhesion [1, 6, 7, 93, 94] (**Figure 1C**).

### Sialic acids as viral receptors

Sialic acids were introduced as the first class of viral receptors, facilitating viral entrance and initiation of infection in a wide range of human and animal host cells [6]. Many viruses such as influenza, parainfluenza, mumps, corona, noro, rota, and DNA tumor viruses bind to sialylated structures like glycoproteins and gangliosides on their host cell membrane [6, 7]. In the viral attachment process, a particular binding site or packet on the viral protein recognizes one or more sialic acid derivatives [8,9,10,11]. Natural sialyl conjugates are α2-3- or α2-6-linked to Gal and GalNAc, 2-6-linked to GlcNAc, or α2-8-linked to the second sialyl residue [12,13,14,15]. Among viral attachment proteins, hemagglutinin is one of the most important on the surface of influenza and coronaviruses [16, 17]. Human influenza viruses predominantly bind to Neu5Acα2-6Gal on epithelial cells in the human airway epithelium [18].

By contrast, avian influenza viruses preferentially recognize Neu5Acα2-3-Gal on the intestinal and respiratory tract cells of birds [14, 15]. Murine coronaviruses recognize 4-*O*-acetyl-*N*-acetylneuraminic acid [19, 20], Sendai virus and human parainfluenza virus 1 (HPIV1) bind to α2-3 linkages [21], and HPIV3 has α2-6 specificity [22]. In addition to viral attachment proteins, viral enzymes such as neuraminidase (sialidase) and esterases, especially acetyl esterases, promote viral release at the end of the life cycle and prevent clumping of virions by removing receptors from the cell surface and viral glycoproteins, respectively [23, 24]. Sialic acids are essential for the entry of coronaviruses to target cells, and these viruses seem to have 2 domains for binding to the host cell [25]. This 2-step process begins primarily with viral binding to sialic acids on the cell surface. This step facilitates adherence to protein receptors and later stages of viral infection [26]. A study of the difference in immune-inflammatory markers between groups of patients with severe acute respiratory syndrome coronavirus 2 (SARS-CoV-2) infection revealed that serum level of sialic acid is markedly higher in severe cases than in those without severe infection [5]. Viruses with specific binding to sialyl derivatives, and their attachment proteins, are listed in **Table 1**.

**Table 1:** Viruses with specific binding to sialic acid derivatives

**Virus name**	**Viral genus**	**Viral family**	**Genome type**	**Envelope**	**Sialic acid receptor**	**Viral attachment protein**	**Host(s)**	**Refs.**
*Influenza A virus*	*Alphainfluenza-virus*	Orthomyxoviridae	ssRNA	Yes	Neu5Acα2-3/6Gal, Neu5Acα23/6GalNAc, Neu5Acα2-6GlcNAc	HA, NA	Birds and mammals	[8, 13,14,15, 95]
*Influenza B virus*	*Betainfluenza-virus*	Orthomyxoviridae	ssRNA	Yes	Neu5Acα2-3/6Gal, Neu5Acα23/6GalNAc, Neu5Acα2-6GlcNAc	HA, NA	Human and seals	[8, 13,14,15, 95]
*Influenza C virus*	*Gammainfluenza-virus*	Orthomyxoviridae	ssRNA	Yes	*N*-Acetyl-9-*O*-acetylneuraminic acid (Neu5,9Ac2)	HEF	Human	[95,96,97]
*Salmon isavirus*	*Isa-virus*	Orthomyxoviridae	ssRNA	Yes	4-*O*-5-*N*-Acetylneuraminic acid (Neu4,5Ac2)	HE	Fish	[95, 98, 99]
TGEV	*Alphacorona-virus*	Coronaviridae	ssRNA	Yes	α2-3-linked *N*-glycolylneuraminic acid	HA	Pigs	[6, 100, 101]
BCoV, HCoVOC43	*Betacorona-virus*	Coronaviridae	ssRNA	Yes	9-*O*-Acetylated sialic acid	HE, S protein	Bovine and Human	[6, 102]
SARS-CoV-2	*Betacorona-virus*	Coronaviridae	ssRNA	Yes	*N*-Acetylneuraminic acid	S protein	Human	[103]
IBV	*Gammacorona-virus*	Coronaviridae	ssRNA	Yes	α-2-3-Linked sialic acid	HA	Birds	[6, 104]
Bovine torovirus	*Toro-virus*	Coronaviridae	ssRNA	Yes	Neu5,9Ac2 and *N*-acetyl-7,9-*O*-acetylneuraminic acid	HE	Bovine	[6, 20]
HPIV1	*Respiro-virus*	Paramyxoviridae	ssRNA	Yes	α-2-3-Linked sialic acid	HA, NA	Human	[6]
Murine norovirus	*Noro-virus*	Caliciviridae	ssRNA	No	Terminal sialic acid in GD1a	VP1	Murine	[105, 106]
Enterovirus 70	*Entero-virus*	Picornaviridae	ssRNA	No	*O*-Linked glycans containing sialic acid α2-3-linked to galactose	(DAF/CD55)	Human	[107, 108]
Human rotavirus	*Rota-virus*	Reoviridae	dsRNA	No	GM1	VP4	Human	[6]
Murine polyomavirus	*Alphapolyoma-virus*	Polyomaviridae	dsDNA	No	Neu5Acα2-3-Gal	VP1	Murine	[6]

Neu5Ac, *N*-acetylneuraminic acid; Gal, galactose; GalNAc, *N*-acetyl galactose; GlcNAc, *N*-acetyl glucose, HA, hemagglutinin; NA, neuraminidase; HEF, hemagglutinin–esterase (acetylesterase)-fusion, HE, hemagglutinin–esterase (acetylesterase); TGEV, transmissible gastroenteritis virus; BCoV, bovine coronavirus; HCoVOC43, human coronavirus OC43; SARS-CoV-2, severe acute respiratory syndrome coronavirus 2; IBV, infectious bronchitis virus; HPIV1, human parainfluenza virus 1; VP1, viral protein 1; VP4, viral protein 4; DAF/CD55, complement regulatory protein decay-accelerating factor; GM1, monosialotetrahexosylganglioside; DAF, decay-accelerating factor.

Therefore, sialic acid derivatives have a critical role in many critical viral infections. Targeting sialic acid production and virus–sialic acid interaction may provide new insights into therapeutic options for viral infections.

### Immune system evasion of cancers through sialic acid modification and potential cancer treatment strategies targeting sialic acids

Upregulation of sialic acids in cancerous cells can play a fundamental role in immune system dampening, progression of tumor growth, and metastasis [27]. This enhanced glycosylation level results in dysregulation of enzymes modifying sialic acids, such as sialyltransferases and sialidases [28]. Sialyltranferases are enzymes that transfer sialic acids to the terminus of glycoconjugates. The overexpression of specific sialyltransferases is correlated with hypersialylation and poor prognosis in cancer progression [29, 30]. Moreover, sialidases can catalyze the removal of sialic acids from glycoconjugates. Hence, decreased expression of sialidases can contribute to increased levels of sialic acids on cell surfaces [31]. Accumulating evidence clarifies the potential role of hypersialylated cell membranes of malignant cells in modulating and evading the immune system. The innate immune system includes lymphocytes known as natural killer (NK) cells, which are critical players in targeting malignant cells by releasing granzymes and perforins. NK cells can also activate the adaptive immune system by releasing various cytokines, including tumor necrosis factor-alpha (TNF-α) and interferon-gamma (IFNγ), which promote T-cell effector functions to eradicate malignant cells [32, 33]. Tumor cells are proposed to evade the immune system through interactions between their hypersialylated membranes and sialic acid-binding immunoglobulin-like lectins (Siglecs) through the sialic acid–Siglec axis. Siglec-7 and Siglec-9 are 2 inhibitory receptors causing cellular inactivation, expressed by NK cells. The dense layer of sialic acids on the surface of tumor cells engages these inhibitory receptors. This modulates the immune system by overall reducing and inhibiting NK cell activities [28, 34,35,36]. As a result, the abnormal glycosylation of tumor cells and subsequent immune system evasion seems to help the tumor to invade local tissue, entering the blood or lymphatic system, with consequent extravasation, leading to metastasis [37].

The high density of sialic acids expressed on cell membranes of cancer cells confers a negative charge on the membrane [38]. This charge inhibits interactions between cells and might facilitate cell detachment from the tumor mass and their subsequent metastasis into the bloodstream [39]. Sialic acids limit the alternative complement pathway by binding to factor H, an essential regulator of the alternative pathway [40]; adding sialic acids to nanoparticles may dampen the innate immune response as suggested because these negatively charged nanoparticles evade being engulfed by macrophages in vitro. Sialic acids inhibit the expression of activated macrophage markers, CD14 and CD86, and cause the macrophages to stay silent and inactivated. Moreover, sialic acid-coated nanoparticles suppressed the formation of markers of inflammation such as TNF-α and IL-6 [41]. Thus, higher levels of sialic acids on tumor cells may dampen the immune system, thereby facilitating metastasis. Targeting the sialic acid-coated cancer cells is a potential therapeutic strategy for cancer treatment. Measuring sialic acid levels in patients with cancer may give us a better understanding of tumor and metastatic progress.

Siglecs are also expressed on cancerous cells and normal immune cells, playing a potentially vital role in dampening the immune response and molding the behavior of tumor cells. The expression of Siglecs on tumor cells is enhanced and mutated in malignancies such as endometrial carcinoma, melanoma, and non-small-cell lung cancer. mRNA levels of Siglec family genes are highly correlated with immune response. In head and neck squamous cell carcinoma, thyroid carcinoma, and thymoma, the expression of Siglec family genes is negatively associated with infiltration of B cells and cytotoxic T cells, while in cervical squamous cell carcinoma, robust expressions of Siglec genes seem to be positively linked with infiltration of adaptive immune cells [42, 43]. Siglec-15 has received substantial attention as a possible pillar of cancer therapy. Siglec-15 is expressed on many malignant cells besides its regular expression in macrophages and myeloid cells. It appears to play a crucial role in suppressing the immune response in the tumor microenvironment (TME). Instead of systemically stimulating the immune system, selective blockade of Siglec-15 by monoclonal antibodies or genetic manipulation of Siglec-15 genes has raised hopes of bringing new insights into selectively targeting cancer cells [44,45,46]. Treatment with anti-Siglec-15 antibody promoted the formation of osteoblasts in cultures of mouse bone marrow cells in the presence of receptor activator of nuclear factor-k B ligand and macrophage colony-stimulating factor [47].

CMP-sialic acid is a putative primary metabolite responsible for the sialylation of cancer cells and causing metastatic breast cancer. To prevent metastatic breast cancer, rather than targeting specific classes of sialyltransferases, a proposed method is to knock down *CMAS*, the underlying gene in the sialic acid pathway [48, 49].

A potential role of immunotherapeutic agents in altering glycosylation in cancer treatment is proposed. The ganglioside GD2 is expressed abundantly in neuroblastomas, in contrast with normal healthy tissues that do not contain high levels of GD2 on their cell surfaces. Immunotherapeutic agents such as a monoclonal anti-GD2 antibody (dinutuximab) induce NK cells to attack neuroblastoma cells by activating the antibody-dependent cell-mediated cytotoxicity (ADCC) pathway. This targeting depends on the recognition of aberrant GD2 sialylation on the malignant cells. Synergistic GD2 upregulation by Ac_5_Neu5Ac, a sialic acid analog, and histone deacetylase inhibitors (HDI) suggested that combined sialic acid analogs and HDIs may boost anti-GD2 monoclonal antibody therapy for neuroblastomas [50, 51]. A further strategy to suppress tumor growth includes blocking sialic acid expression of malignant cells by injecting a fluorinated sialic acid mimetic into the tumor bulk. Blocking sialic acid expression by neuroblastoma and melanoma cells by intratumoral injection of Ac_5_3F_ax_Neu5Ac, a fluorinated sialic acid derivative, attenuated the tumor growth in vivo by the direct inhibition of sialyltransferases. The dense layer of sialic acids on the malignant cell membranes helps the tumor evade the immune system and causes metastasis formation. Ac_5_3F_ax_Neu5Ac can potentiate the immune response in the TME by inducing activated immune cells, including dendritic cells and CD8^+^ cells, and by decreasing the number of regulatory cells such as myeloid regulatory cells and regulatory T cells [52].

Another novel immunotherapeutic strategy targets the CD24-Siglec10 signaling pathway through anti-CD24 monoclonal antibodies or genetic ablation of Siglec-10 genes. Several neoplasms express an extensive amount of CD24. Upregulated expression of CD24 on cancer cells is closely related to the prognosis of ovarian cancer [53] and triple-negative breast cancer [54]. A higher expression of CD24 in cancerous cells than in other cell clusters has proposed CD24 as a tumor-specific antigen in the mentioned malignancies. Moreover, tumor-associated macrophages (TAM) appear to express Siglec-10 genes profoundly, proposing the CD24-Siglec10 inhibitory signaling cascade as a potential strategy for cancers to evade inflammatory responses efficiently. It has been shown that blocking the CD24-Siglec10 signaling pathway components by monoclonal antibodies against CD24 and Siglec10 or genetic ablation of involved genes can robustly increase the phagocytic activity by TAM and destroy malignant cells by enhancing immune responses in the TME [55]. Overall, studies have delineated the potential role of exploiting sialic acids by tumors to dampen inflammatory responses and progress and form metastasis. Thus, targeting signaling mechanisms involved in this process through genetic ablation and monoclonal antibodies is a potential strategy for cancer treatment. This idea should encourage future studies to target sialic acids and the involved signaling cascades as the leading players in cancer progression.

### Sialic acids and CVD

Coronary artery disease (CAD) is the most prevalent cause of death globally. Inflammation, fibrinolysis, and oxidative stress seem to be the leading causes of plaque disruption and subsequently lead to CAD. Thus, markers that participate in these pathophysiological pathways may be used as prognostic factors [5, 56]. Sialic acids, which are also expressed on circulatory molecules, may affect the pathogenesis, progression, predictions, and severity of the atherosclerotic process and CVD [57].

Raised serum sialic acid levels in patients with CAD compared with healthy controls has a significant correlation with CVD risk factors. Therefore, serum sialic acid levels may be considered a strong predictor of CVD severity and mortality [56, 58]. Sialic acid is also a marker of vascular involvement in the atherosclerotic processes, probably related to an inflammatory response of the arterial wall [58, 59]. Zhang et al. analyzed the plasma metabolites in 2324 patients who had coronary angiography, and they confirmed a significant increase in Neu5Ac plasma levels correlated with CAD progression. They also performed a study in vitro. They found that administration of Neu5Ac led to increased levels of lactate dehydrogenase and creatine kinase-MB and apoptosis in neonatal rat ventricular myocytes. Their results suggest that the administration of neuraminidase-1 inhibitors, including oseltamivir and zanamivir, as used against the influenza virus, can act as cardioprotective agents, potentially preventing CAD progression and myocardial injury [60]. The mechanism involved in the rise in serum sialic acid among CVD patients may be various, including oxidative stress and inflammation, which can act in atherosclerosis and CVD [61].

Sialic acid is associated with the formation of inflammatory cytokines such as interleukin-6 (IL-6) and TNF-α [62, 63]. There is a significant increase in acute-phase reactants containing sialic acids. Therefore, like C-reactive protein (CRP), sialic acid could be used as an associated factor to predict CVD. Sialic acid has not only been suggested as the most stable acute-phase marker, but it has also been proposed as a suitable marker of the overall acute-phase response in indicating CVD [62, 64]. Moreover, due to the increased sialylation of plasma proteins during an inflammatory response, protein-bound sialic acid increases and reflects the clustering of cardiovascular risk factors. Sialic acid could scavenge reactive oxygen species (ROS), which subsequently prevent alteration of biological macromolecules, such as desialylation, which is a possible primary step of atherogenic modification. This occurs by the glycosidic bonding of sialic acid as a potential target for superoxide and other related ROS [65]. Desialylation may lead to cellular dysfunction and uptake of circulatory sialoglycoprotein by the hepatocyte sialoglycoprotein receptor. Remarkably, although CVD progression is accompanied by desialylation in glycoconjugates, serum total sialic acid (TSia) seems to rise [66]. Moreover, sialic acid-free low-density lipoprotein (LDL) tends to increase and accumulate in endothelium and smooth muscle cells, suggesting that low sialic acid content of LDLs may be atherogenic. Thus, it can be assumed that increased sialic acid levels can be a risk factor for CVD unless the levels reflect LDL-unbound sialic acid [66, 67]. CVD is a leading cause of morbidity and mortality worldwide, and there is also an increased incidence of its risk factors. Some clinical and epidemiological studies of CVD have shown that sialic acid has the best sensitivity and specificity and produces the least number of false negatives and false positives of other sensitive biomarkers such as highly sensitive CRP, oxidation of LDL, malondialdehyde, plasminogen activator inhibitor, and vitronectin, suggesting the value of sialic acid as a biomarker in clinical monitoring. Serum levels are significantly higher in patients with CAD than in healthy controls [56].

### Sialic acid as a predictor of diabetic complications

**Figure 2:**
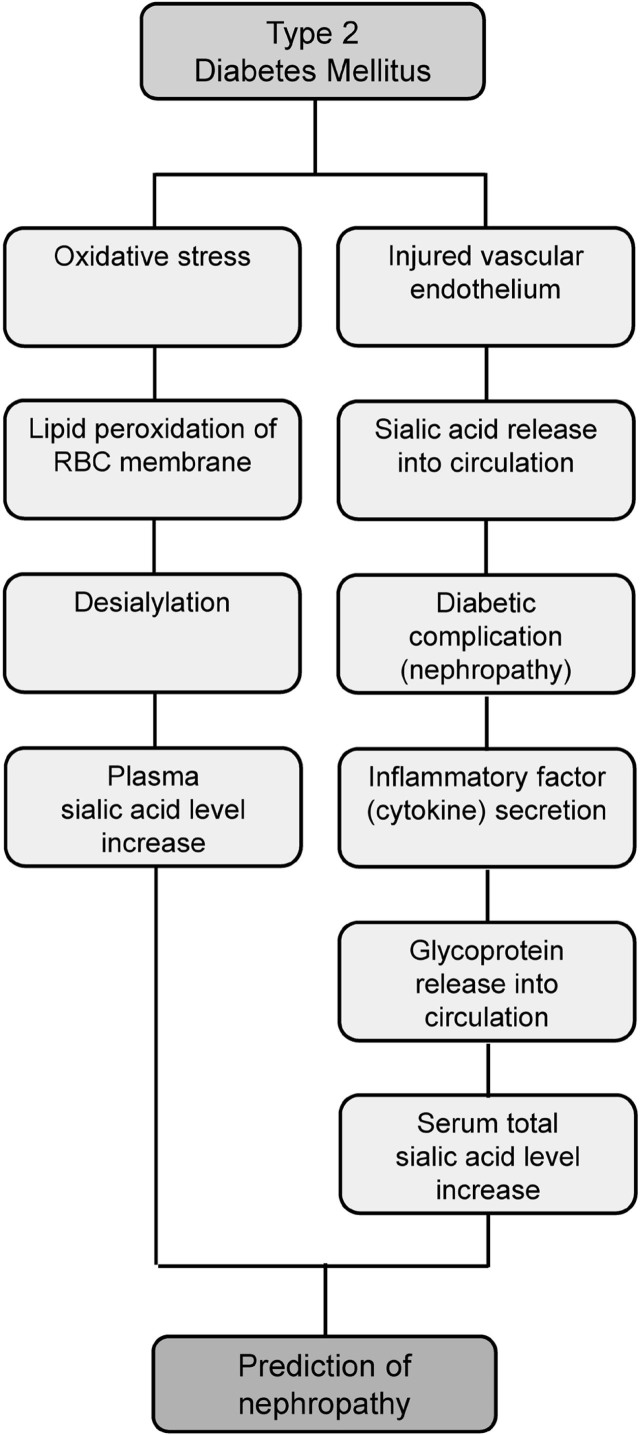
Flowchart showing that increased sialic acid level in plasma predicts diabetic nephropathy. RBC, red blood cell.

The serum TSia level and plasma sialic acid (PSia) are increased to more than the normal range in type 2 diabetic (T2D) patients. TSia level in patients with diabetic nephropathy rises significantly compared with non-nephropathic patients [4, 68,69,70,71]. High concentrations of sialic acid derivatives are found in the vascular endothelium of the retina, kidneys, heart, and brain. Consequently, micro- and macrovascular complications in patients with T2D lead to increased TSia levels, and this causes retinopathy, nephropathy, and neuropathy [68, 71]. Furthermore, infiltrating immune cells such as macrophages and endothelial cells produce inflammatory factors such as cytokines in injured tissues. Next, the acute-phase response stimulates the secretion of glycoproteins containing sialic acids from the liver into the blood circulation, leading to increased TSia level and urinary excretion of sialic acid [68, 69, 72]. Oxidative stress during diabetic nephropathy causes lipid peroxidation of the red blood cell membranes, followed by desialylation. Removal of sialic acid moieties from the erythrocyte membranes increases PSia level and causes cell aggregation [73, 74]. Overall, TSia and PSia levels may provide valuable risk factors to predict diabetes-induced nephropathy early and may help to manage diabetic patients (**Figure 2**).

### Role of sialic acids in the central nervous system

Glycosylation is essential for brain activity, and any change in this process can induce nervous system disorders [75]. Sialylation is a type of glycosylation abundantly found in brain tissue. Siglecs are expressed on the surface of neural cells and play important roles in neuroinflammation [76]. Sialoglycans are essential for the development of the nervous system. There are 2 families of sialoglycans in the brain: gangliosides and polysialic acid. Gangliosides are the most common form of sialoglycans in neurons. They participate in interactions between axons and myelin, regeneration of axons, and the excitability of nerve cells.

Polysialic acid modulates interactions between cells, matrix, and different molecules in the cellular environment. It is involved in brain development, neuroplasticity, and polymorphisms [77]. There is a correlation between psychiatric disorders such as schizophrenia, autism, and bipolar disorder and the genes responsible for synthesizing polysialic acid [77]. Neurological disorders affect sialylation during the inflammatory reaction of microglia. Chronic stress seems to lower the sialidase activity, which is associated with increased polysialic acid expression [78]. Sialidase enzymatic activity changes during epileptic seizures in the brain [79], and memory processing is based on the negative charge of the sialic acids expressed on the cellular membrane [80]. Sialidase prevents the release of glutamate from hippocampal neurons and synaptic vesicles by regulating calcium signaling during neurotransmission [81]. Deficiencies of lysosomal sialidases can cause the accumulation and amyloidogenic processing of an oversialylated amyloid precursor protein, and extracellular release of neurotoxic amyloid-β peptides, which are related to the pathogenesis of Alzheimer disease [82]. Sialidases may enhance GM1 ganglioside expression and be neuroprotective in a murine model of Parkinson disease [83]. Finding altered sialylation and sialidase activity in nervous system disorders may encourage future studies to elucidate the pathophysiology of sialic acids in neurological disorders and identify therapeutic drugs targeting these activities.

## Sialic acids as promising targets for novel therapeutic drugs

Angiogenesis plays an essential role in developing various diseases, including neoplasms [84]. Sialic acids are widely expressed in endothelial cells, and they are linked with various gangliosides involved in angiogenesis and neovascularization. Thus, sialic acids may be used to develop anti-angiogenic strategies for neoplasms. These strategies include inhibiting sialidases, sialyltransferases, and enzymes involved in the synthesis of gangliosides, complete desialylation of the endothelial cells, and removal of sialic acid-mimicking compounds leading to the formation of angiogenic growth factors [84,85,86]. The chemotherapeutic agent, etoposide, has limited applications because of its severe adverse effects and poor solubility. Inhalable aminophenyl boronic acid–human serum albumin nanocomposites have provided a technique for the systemic delivery of etoposide to treat lung cancer. Intratumoral transport of aminophenyl boronic acid could be facilitated by binding to sialic acids. Hence, its tumor-targeting effect and cytotoxicity are improved against lung cancer cells compared with untargeted nanoparticles or free drugs [87]. Phenyl boronic acid arrangement could help nanoparticles target malignant cells more efficiently and extend the time they are retained in the TME due to the interaction between hypersialylated cancer cells and phenylboronic acid groups [88, 89]. The importance of finding suitable sites for modifying sialic acid, changing drugs/carrier linkage, and optimizing the density of sialic acid modified in the drug-delivery systems is emphasized [89]. Quercetin 7*-O*-sialic acid (QA) may outperform quercetin in preventing oxidative and inflammatory processes, promoting cholesterol efflux, and protecting molecules against the desialylation process. Moreover, QA significantly decreases the expression of TNF-α, monocyte chemoattractant protein-1, intercellular adhesion molecule-1 (ICAM-1), and vascular cell adhesion molecule-1. The addition of *N*-acetylneuraminic acid to quercetin increases the solubility of quercetin by adding carboxyl groups, which may increase the possibility of potentially using QA for therapeutic approaches and has therefore been proposed to prevent or treat CVD [90]. Modification of sialic acids by gold nanoparticles may allow them to evade the reticuloendothelial system by inhibiting immune responses and increasing tumor accumulation via its active targeting ability [42]. Sialic acid-based micelles with bone repairing activities have the potential to treat rheumatoid arthritis. Methotrexate treatment of arthritis is associated with high toxicity and serious adverse effects, and inhibits bone regeneration on previously formed erosions in patients. A sialic acid-dextran-octadecanoic acid (SA-Dex-OA) conjugate was synthesized to form micelles. The conjugate dramatically corrected aggregation and transportation by combining sialic acid and E-selectin receptors in inflamed cells. MTX-loaded SA-Dex-OA micelles have been shown to suppress the inflammatory response, in addition to decreasing the adverse effects of MTX [91]. Doxorubicin-loaded SA-Dex-OA micelles have been proposed to target hepatocellular carcinoma. SA-Dex-OA micelles have multifold accumulation in TMEs and mediate doxorubicin delivery into tumor cells more effectively than micelles without sialic acid modification. Thus, sialic acid-functionalized micelles with double targeting effects have the potential for liver cancer treatment [92].

Sialylation promotes amyloid-β interaction, internalization, and transportation to the brain. Selective desialylation of amyloid-β is a potential target for sialidases as potential therapeutic agents for Alzheimer disease [83].

The essential role of sialic acids in coronavirus infections has been highlighted [26]. Blocking sialic acids or desialylation of cell membranes may be a therapeutic target in preventing and treating coronavirus infections. Exploiting sialic acid modification in different diseases and cancers seems to be a promising strategy for novel drugs designed with nanotechnology. Drugs, potential therapeutic targets, therapeutic effects, and mechanism of action of sialic acid in various diseases are listed in **Table 2**.

**Table 2:** Potential therapeutic targets and effects and mechanism of action of sialic acid in various diseases

**Disease**	**Drug or potential therapeutic target**	**Therapeutic effects**	**Mechanism of action**	**Refs.**
Alzheimer disease	Human β-amyloid SA-binding lectin LFA	Decrease in amyloid plaque deposition in the brain, reducing neuroinflammation	Binding to gangliosides in neuronal lipid rafts and inhibiting the binding of amyloid peptides	[109]
CVD	Quercetin 7-*O*-SA Neuraminidase inhibitors (oseltamivir and zanamivir)	Antioxidation, antiinflammation, cholesterol efflux, promotion of biomolecule protection, prevention of CAD progression	Antioxidant, antiatherosclerosis, decreased hydrogen peroxide, reduced expression of TNF-α, MCP-1, ICAM-1, VCAM-1, reducing Neu5Ac levels by inhibiting its regulatory enzyme (neuraminidase)	[90, 60]
Cancer	SA/mPEG AuNPs EPI-SL PSA-TPGS SA-imprinted BS-NPs Blockade of CD24-Siglec-10 interaction in TNBC and ovarian cancer Combination of sialic acid analog, HDI, and anti-GD2 antibody (dinutuximab) in neuroblastoma Fluorinated sialic acids (e.g., Ac5_3_F_ax_ Neu5Ac)	Inhibiting the evasion of the immune response, improvement of the tumor-targeting efficiency and antitumor activity, improvement in immune responses, enhancement of cell cytotoxicity, clearance of cancer cells by macrophages, localized blockade of tumor antigens	Evasion of the reticuloendothelial system Enhancement endocytosis of liposomes by circulating N/Ms ABC phenomenon weakness Cleavage of intracellular GSH Enhancement of phagocytic activity using monoclonal antibodies targeting CD24-Siglec-10 interaction in ovarian cancer and TNBC Concurrent increase of sialyltransferase expression involved in GD2 synthesis and blockade of GD2 in neuroblastoma Repeated injection of fluorinated sialic acids can directly inhibit tumor-associated sialyltransferases in melanoma and neuroblastoma	[41, 50, 52, 55, 110, 111]
Viral infection (chicken new castle, influenza virus, coronavirus, and rotavirus)	Ovomucin Oseltamivir	Antiviral activity	Unknown Neuraminidase inhibitors	[93, 112]
Neurological Disorders	SAPPM copolymer for delivering DDS in SCI Ac3ManNAc for delivering ManNAc-6-P in GNE myopathy	Neuroregeneration, increased neuroprotective capacity, providing substrate for sialic acid biosynthesis	Enhancing the distribution of DDS via binding to E-selectin in SCI Increase in sialic acid levels via providing the deficient substrate in GNE (IBM2) myopathy	[76, 113, 114]
Rheumatoid arthritis and comorbid tumors	Sialic acid-modified doxorubicin hydrochloride liposome (DOX-SAL)	Accumulation reduction of inflammatory neutrophils at the disease site	Induction of PBN apoptosis by binding to L-selectin	[115]

SA, sialic acid; LFA, *Limacus flavus* agglutinin; CAD, coronary artery disease; CVD, cardiovascular diseases; TNF-α, tumor necrosis factor-α; MCP-1, monocyte chemoattractant protein-1; ICAM-1, intercellular adhesion molecule-1; VCAM-1, vascular cell adhesion molecule-1; mPEG, methoxy polyethylene glycol; AuNPs, gold nanoparticles; EPI-SL, SA-stearic acid conjugates and epirubicin-loaded liposomes; N/Ms, neutrophils and monocytes; PSA-TPGS, polysialic acid and d-α-tocopheryl polyethylene glycol 1000 succinate conjugates; ABC, accelerated blood clearance; BS-NPs, biodegradable silica nanoparticles; GSH, glutathione; Siglec-10, sialic acid-binding immunoglobulin-type lectins; TNBC, triple-negative breast cancer; SAPPM, sialic acid-polyethylene glycolpoly (lactic-co-glycolic acid); DDS, drug-delivery system; SCI, spinal cord injury; Ac3ManNAc, 1,3,4-*O*-acetylated *N*-acetylmannosamine; ManNAc-6-P, *N*-acetylmannosamine 6-phosphate, PBN, peripheral blood neutrophil; HDI, histone deacetylase inhibitors.

Targeted drug-delivery systems may play a vital role in therapy for diseases and cancers by causing fewer adverse effects and enhanced antitumor activity than traditional medications.

## Tissue specificity of sialic acids in various diseases

Any disease that causes vascular damage can elevate the sialic acid level. Correlation between different conditions such as viral infections, nervous system disorders, cancers, thromboembolism, diabetic nephropathy, and increased sialic acid levels has been found [5, 68]. Free and bound sialic acid (TSia) has been measured in serum, plasma, and tissue homogenates by various methods (**Table 3**). Most of these methods are research methods, and among them, enzyme-linked immunosorbent assay (ELISA) and high-performance liquid chromatography (HPLC) are more specific and more accurate than others and may be used in clinical applications. There are diagnostic parameters for sialic acids based on target disease and tissue (**Table 4**). However, the level of total serum sialic acid seems to be an unspecific and general marker, and it can increase in a variety of conditions.

**Table 3:** Various methods for sialic acid detection

**Method**	**Example**	**Advantages**	**Disadvantages**	**Refs.**
Biochemical assay	Resorcinol assay	Assays bound and free SA	Considerable interferences by pentoses, hexoses, and uronic acids	[116]
	Periodate–thiobarbituric acid assay	Assays free SA	Considerable interferences by 2-deoxyribose, unsaturated fatty acids, lactose, and maltose Bound SA not detected	[117]
	Roboz assay	Assays bound and free SA Eliminates interferences such as deoxyribose, fatty acids, and some neutral carbohydrates	Slight turbidity in the assay solution	[118]
	Periodic acid/MBTH	Assays total SA without release sialic acids by acid hydrolysis or neuraminidase treatment,	Considerable interferences	[119, 120]
	Acidic ninhydrin assay	Assays bound and free SA	Considerable interferences	[121, 122]
Enzymatic and fluorometric assay		Measurement total		[123]
		Neu5Ac		
		Simple and convenient		
		No significant cross-reactivity or interference		
Enzymatic and calorimetric assay		Measurement total		[123,124,125]
		Neu5Ac		
		Simple and convenient		
		No significant cross-reactivity or interference		
HPLC fluorescent detection		High specificity	Expensive, research use only	[125]
ELISA and calorimetric assay		High specificity	Research use only	[56]

SA, sialic acid; MBTH, 3-methyl-2-benzothiazolone hydrazone assay, HPLC, high-performance liquid chromatography; ELISA, enzyme-linked immunosorbentassay.

**Table 4:** Diagnostic parameters for sialic acid

**Target disease**	**Detection methods for SA**	**Target tissue**	**Sensitivity (%)**	**Specificity (%)**	**Cut-off**	**AUC (%)**	**Refs.**
Breast cancer	Surface-enhanced Raman spectroscopy	Saliva	80	93	12 mg/dL	95	[127]
Breast cancer	Surface-enhanced Raman spectroscopy	Saliva	80	100	15.5 mg/dL	94.05	[128]
Rheumatoid arthritis	HPLC	Serum	84.2	92	766.70 μg/mL	92.1	[126]
Nasopharynx cancer	Enzymatic method	Serum	30.95	83.33	≥650 mg/dL	80.7	[129]
COVID-19 and gastrointestinal tract	ELISA	Serum	76.2	73.7	74.55 mg/dL	84	[130]
Oral squamous cell carcinoma	Spectrophotometric method	Saliva	100	100	>0.30 μg/mL	100	[131]
		Blood	93.33	100	>4.23 μg/mL	98.3	

SA, sialic acid; AUC, area under the receiver operating characteristic curve; HPLC, high-performance liquid chromatography; COVID-19; coronavirus disease 2019; ELISA, enzyme-linked immunosorbent assay.

Measuring tissue-specific sialic acids may provide new insights into distinguishing the origin of elevated sialic acids in the serum [3]. The possibility of tissue specificity of sialic acids warrants further investigation.

## Conclusions

Sialic acids may play an important role in the pathophysiology of many diseases, including infection, malignancy, CVD, neurological disorders, and diabetic nephropathy. Abnormal overexpression of sialic acids on malignant cells and their interaction with immune cells seem to allow tumors to evade the immune system. However, routine measurement of serum sialic acid is not recommended as a classic marker of inflammation, although serum sialic acid may be better than measuring other acute-phase reactants, including CRP. We suggest that serum sialic acid level could be employed as a general and routine marker of severity in various diseases, especially neoplasms and infection; tissue specificity of sialic acids may suggest the organs involved. The potential of sialic acid as a crucial routine marker in different pathological conditions warrants exploration. Sialic acid and associated enzymes are promising therapeutic targets for improving anticancer and antiviral drugs and medications for CVD, neurological disorders, and diabetic complications.
